# Differential Gene Expression following *DHX36*/*G4R1* Knockout Is Associated with G-Quadruplex Content and Cancer

**DOI:** 10.3390/ijms25031753

**Published:** 2024-02-01

**Authors:** Joseph M. Gumina, Adam E. Richardson, Mahmudul Hasan Shojiv, Antonio E. Chambers, Siara N. Sandwith, Michael A. Reisinger, Taylor J. Karns, Tyler L. Osborne, Hasna N. Alashi, Quinn T. Anderson, Meredith E. Sharlow, Dylan C. Seiler, Evan M. Rogers, Anna R. Bartosik, Melissa A. Smaldino, James P. Vaughn, Yuh-Hwa Wang, Philip J. Smaldino, Robert A. Haney

**Affiliations:** 1 Department of Biology, Ball State University, Muncie, IN 47306, USA; 2 School of Medicine, University of Virginia, Charlottesville, VA 22908, USA; 3 NanoMedica LLC, Winston-Salem, NC 27106, USA

**Keywords:** DHX36, G4R1, RHAU, G-quadruplex, helicase, leukemia, Jurkat cells

## Abstract

G-quadruplexes (G4s) are secondary DNA and RNA structures stabilized by positive cations in a central channel formed by stacked tetrads of Hoogsteen base-paired guanines. G4s form from G-rich sequences across the genome, whose biased distribution in regulatory regions points towards a gene-regulatory role. G4s can themselves be regulated by helicases, such as DHX36 (aliases: G4R1 and RHAU), which possess the necessary activity to resolve these stable structures. G4s have been shown to both positively and negatively regulate gene expression when stabilized by ligands, or through the loss of helicase activity. Using *DHX36* knockout Jurkat cell lines, we identified widespread, although often subtle, effects on gene expression that are associated with the presence or number of observed G-quadruplexes in promoters or gene regions. Genes that significantly change their expression, particularly those that show a significant increase in RNA abundance under *DHX36* knockout, are associated with a range of cellular functions and processes, including numerous transcription factors and oncogenes, and are linked to several cancers. Our work highlights the direct and indirect role of DHX36 in the transcriptome of T-lymphocyte leukemia cells and the potential for DHX36 dysregulation in cancer.

## 1. Introduction

G-quadruplexes (G4s) are four-stranded nucleic acid secondary structures composed of four guanine bases oriented in stacks of planar arrays that form under physiological conditions [1,2]. Each participating guanine interacts with two neighboring guanines via Hoogsteen base pairing to stabilize the G4 structure. G4s are further stabilized by a monovalent cation located in a central G4 cavity [3]. G4s are highly stable structures and have been confirmed to form in vitro and in vivo upon the disruption of Watson–Crick base pairings, a necessary occurrence during transcription and replication [1,2,3,4]. 

Guanine-rich DNA with G4-forming potential, in particular sequences with at least three consecutive guanine nucleotides followed by short runs of unconstrained nucleotide composition, is widespread in bacterial and eukaryotic genomes [5]. There is a substantial number of possible alternative G4 structural configurations. For example, G4s can vary in loop length, location, and compositional origin. The DNA quadruplexes can have parallel or antiparallel topology in either strand of DNA, in an inter- or intra-molecular fashion, or between bases in syn- or anti- conformations [1,2,3,6,7]. G4s function in a diverse array of cellular processes, including telomere maintenance, DNA replication, recombination, transcription, and translation [2,8]. Their stable formation, coupled with their resolvability, suggests that cellular mechanisms now known to include helicase enzymes exist to regulate the formation and dissolution of G4s [9,10,11]. 

### 1.1. Evidence for a Role of G4s in Gene Regulation

There are several lines of evidence indicating the involvement of G4s in transcriptional processes and gene regulatory control. The locations of G4s appear to coincide with regions of nucleosome depletion [12,13], a feature typically associated with areas of active transcription, including promoters and enhancers. Moreover, genes in which G4 enrichment/nucleosome depletion are observed are associated with distinct cellular functions, including apoptosis and cell signaling, as well as tissue specificity [14,15]. G4s are also significantly enriched in transcription-factor binding sites and the promoter region of genes, especially in proto-oncogene promoters. More than 40% of all documented genes in the human genome contain at least one promoter G4-forming sequence, suggesting that G4 modulation may provide a significant axis of transcriptional regulation [6,16,17,18,19,20,21].

### 1.2. Potential Effects of G4s on Transcription

The mode of regulatory control exerted by G4s varies depending on the proposed direct, indirect, or epigenetic mechanisms [1,4,22]. The binding of transcription factors or other proteins to regulatory sequence G4s enhances the transcription of certain genes. Moreover, the binding and stabilization of G4s on the non-template strand prevents the reannealing of DNA or interferes with suppressor binding, which facilitate the initiation of repeated transcription [1,23,24]. Alternatively, the formation of G4s in regulatory regions could have negative effects on target gene transcription. For example, G4s may block the basic transcription machinery, or provide a binding site for suppressive transcription factors at a silencer element [6,23]. Furthermore, G4s may indirectly alter gene expression by acting as a competitive binding site to sequester transcription factors from other consensus sequences [25], or by binding chromatin modifying enzymes to facilitate epigenetic regulation [23]. Together, these observations exemplify the varied and context-dependent regulatory roles G4s can play in either positively or negatively regulating transcription.

### 1.3. G4 Binding and Stabilization by Ligands Can Alter Gene Expression

The treatment of yeast cells with the G4-stabilizer, N-methyl mesoporphyrin IX, leads to the upregulation of genes with potential G4-forming sequences in their promoter regions, supporting a role for G4s in the facilitation of gene expression [19]. Furthermore, multiple studies in human cells show that genes with promoters containing G4 sequences are dysregulated after G4 stabilization with ligands. Expression of the *c-myc* gene, which contains G4s in the promoter region, was downregulated by the ligand stabilization of G4, indicating the action of G4s in transcriptional repression [26,27], while G4 stabilization by pyridostatin significantly altered the expression of eight genes with proximate G4s relative to the control [28], with both up- and downregulation being observed. 

Moreover, the genome-wide targeting of G4s in a human cell line with a G4-selective antibody showed significant G4-mediated alterations in expression, with 1767 protein-coding genes that were differentially expressed relative to control lines in a microarray analysis, with both up- and downregulation again apparent, and with affected gene promoters having higher numbers of G4-forming sequences, particularly upregulated genes [22]. 

### 1.4. Helicases Modulate G4 Effects on Expression

The unwinding of G4 structures in cells is mediated by helicase enzymes that have a potentially important role in G4-mediated gene regulation. This role for G4 helicases is supported by studies in mutant cells that lack the activity of a specific helicase. For example, alterations in gene expression were observed by microarray analysis in yeast mutants lacking activity of the RecQ family helicase Sgs1p, and coding regions with associated G4 motifs were more likely to be downregulated [19,29]. Fibroblasts from Bloom and Werner syndrome patients, lacking activity for RecQ helicases BLM or WRN, showed broad gene expression differences in comparison to control human fibroblasts, as assessed by microarray [30,31]. 

DHX36 (aliases: G4R1 and RHAU) is a DEAH-box helicase produced by the human *DHX36* gene, which has the highest known affinity for both RNA and DNA G4s [7,10,32,33,34]. Given that this enzyme constitutes a critical G4-resolving factor in human cells, its effects on gene expression may be widespread, leading to the significant upregulation or downregulation of target genes in cells lacking DHX36 activity. Consistent with this prediction, DHX36 knockdown in human cell lines reduced the reporter and endogenous expression of the G4-containing gene, *YY1* [35]. Furthermore, knockout of *DHX36* led to a significant change in the abundance of mRNAs, which was dependent on the number of DHX36 binding sites on the mRNA, especially in the 5′ UTR [36]. Another study found that DHX36 associates with polysomes and the reduction in DHX36 increases the translation of repressive G4-containing upstream open reading frames (uORFs) while reducing protein production from the associated coding regions, especially impacting proto-oncogenes, transcription factors, and other epigenetic regulators [37].

Here, we describe the role of the DHX36 in modulating transcription in an immortalized human T lymphocyte Jurkat cancer cell line using RNA sequencing (RNA-seq) by exploring the functional consequences of gene dysregulation related to the loss of DHX36 with bioinformatic analyses. We further explored the association between gene expression and the presence and number of G4s in promoters and gene regions. Our results indicate that the depletion of DHX36 leads to widespread gene expression changes associated with the G4 content of differentially expressed genes. 

## 2. Results

### 2.1. Data Features

A total of 160,544,634 paired-end reads remained after filtering, with paired-end read counts per replicate library ranging from 24,430,301 to 28,216,080. Mapping rates to the human reference genome were uniformly high (Table 1), and most reads were mapped to unique locations, with 84% of reads mapping to annotated exons, 11.6% to introns, and 4.4% to intergenic regions.

### 2.2. Differential Expression

**Figure 1:**
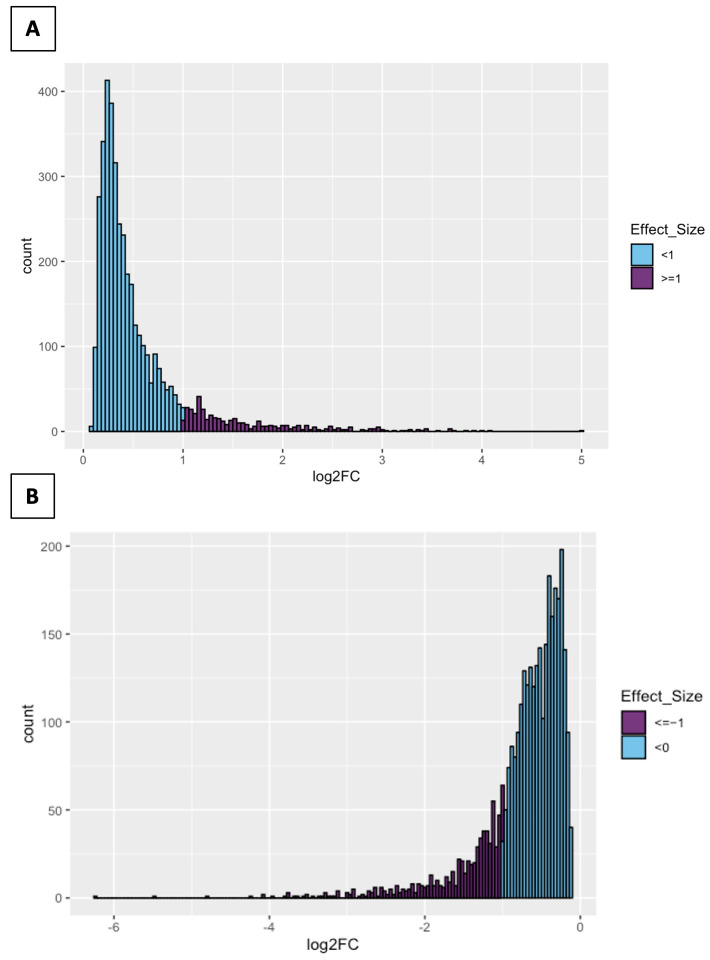
Distributions of effect sizes for genomic features that are upregulated (**A**) or downregulated (**B**) in *DHX36* knockout cell lines relative to control.

A total of 7410 annotated genomic features showed significant differential expression between *DHX36* knockout and control cell lines, with 4026 being upregulated and 3384 downregulated in knockout cell lines. Among the total features that were differentially expressed, 6705 were annotated as protein-coding genes, and 169 as long non-coding RNAs (Supplementary Table S1). Although significant, changes in expression were generally subtle, as only 455 genomic features (of which 384 were protein-coding and 29 were long non-coding RNAs) showed an upregulation of 2-fold or greater (Figure 1) in *DHX36* knockout cell lines, and 675 showed a downregulation of 2-fold or greater (of which 590 were protein-coding and 23 were long non-coding RNAs). The number of genes at higher-level fold changes was limited: 67 were upregulated and 85 downregulated at a fold-change of 5, and 16 were upregulated and 19 downregulated at a fold-change of 10 (Supplementary Table S1). 

A total of 269 differentially expressed genes encoded transcription factors (Supplementary Table S2), of which 173 were upregulated (17 at fold-change of 2 or greater) and 96 downregulated (22 at fold-change of at least -2) in KO cell lines. A total of 362 differentially expressed genes were categorized as oncogenes in the COSMIC database, of which 251 were upregulated (24 at fold-change of 2 or greater) and 111 downregulated (30 at fold-change of at least -2) in KO cells (Supplementary Table S2). Eleven known G4 helicases other than DHX36 [38] were found among the set of significantly upregulated genes, including DDX1, DDX5, DDX21, DDX24, DDX42, BLM, DHX9, DHX11, DNA2, MOV1, and WRN (Supplementary Table S1).

### 2.3. Functional Genomics

**Figure 2:**
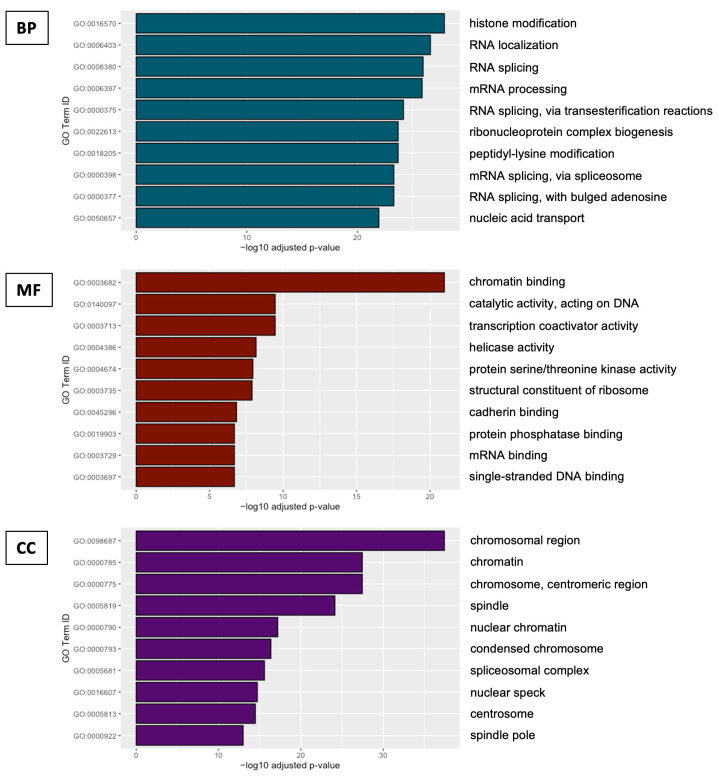
Top 10 most significantly enriched (by adjusted *p*-value) GO terms among three categories for genes upregulated in *DHX36* knockout cell lines. BP = biological process; MF = molecular function; CC = cellular component.

**Figure 3:**
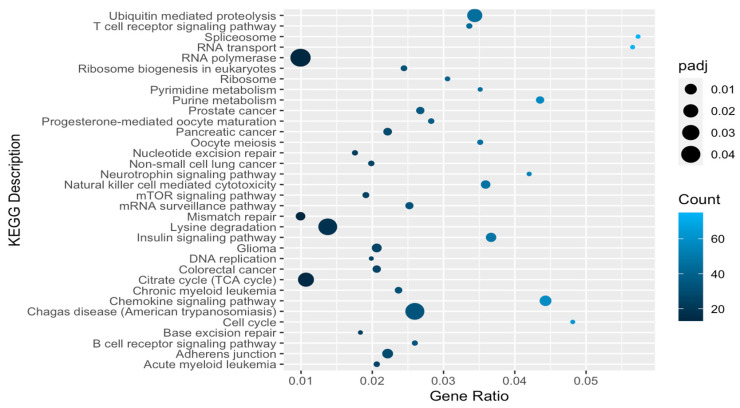
Significantly enriched KEGG categories for genes upregulated in knockout versus control cells. Gene Ratio = fraction of differentially expressed genes matching KEGG description. padj = Benjamini–Hochberg-adjusted *p*-value.

A total of 1280 gene ontology (GO) terms were significantly enriched in knockout line upregulated genes, of which 916 belonged to the biological process category, 157 to the molecular function category, and 207 to the cellular component category (Supplementary Table S3). The most enriched biological process terms related to the processing and transport of RNA, and the most enriched molecular function terms related to nucleic acid and protein binding and modification, helicase activity, and transcriptional coactivation, while enriched cellular component terms included a number related to chromatin and chromosomal structures (Figure 2). In contrast, only 63 GO terms were significantly enriched in *DHX36* knockout-cell-line-downregulated genes, of which 18 belonged to the biological process category and 45 to the cellular component category (Supplementary Table S3), including a number of terms related to cell–cell junctions and vesicle organization and transport. A total of 34 significantly enriched KEGG categories were found for genes that were upregulated in knockout cell lines (Figure 3; Supplementary Table S4), including those related to cell signaling, RNA processing, DNA repair, and several cancers. Only four significant KEGG categories were found for KO-downregulated genes, including lysosome, arrhythmogenic right ventricular cardiomyopathy, axon guidance, and cell adhesion pathways.

### 2.4. Disease Associations of Differentially Expressed Genes

**Figure 4:**
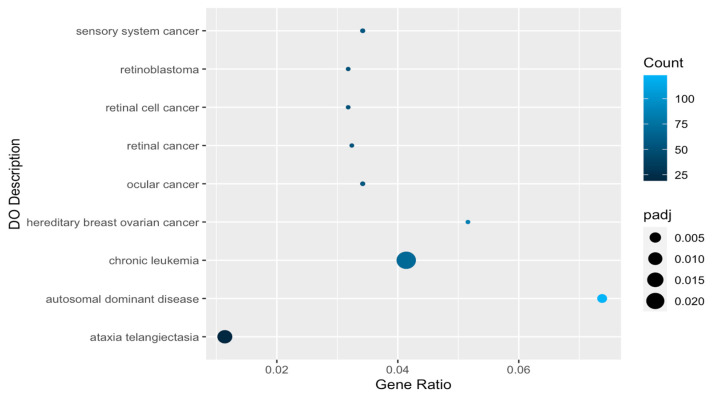
Significantly enriched disease ontology (DO) categories for genes upregulated in knockout versus control cells. Gene Ratio = fraction of differentially expressed genes matching disease ontology description. padj = Benjamini–Hochberg-adjusted *p*-value.

While there were no enriched disease ontology terms for knockout-line-downregulated genes, there were nine for knockout-line-upregulated genes, of which seven were related to ocular or retinal cancers, breast and ovarian cancer, or leukemia (Table 2, Figure 4). The other two significant disease ontology terms were for autosomal dominant disorders in general and the neurodegenerative disorder ataxia telangiectasia.

### 2.5. Differential Expression on DHX36 Knockout Is Associated with G-Quadruplex Content in Promoters

We defined 48,149 promoters as regions directly upstream of the annotated transcription start site for each protein-coding or non-coding gene. A total of 409,365 G4s were consistently identified by G4-Seq in both K^+^ ion and pyridostatin stabilization experiments [39], promoters from a total of 11,358 genes (23.6%) contained at least one observed G4 (OQ), and a total of 17,565 promoter-located OQs were identified for an average of 1.55 OQs per OQ-containing promoter. 

**Figure 5:**
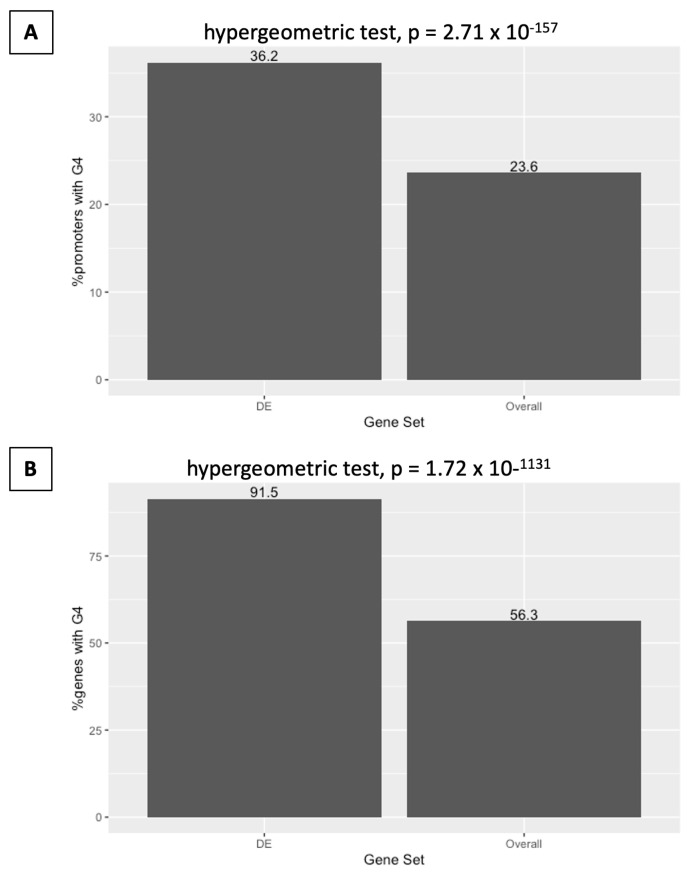
Differentially expressed promoters and genes following *DHX36* KO are more likely to contain OQs compared to the genomic background. (**A**) Percentage of gene promoters with OQ. (**B**) Percentage of gene regions with OQs.

**Figure 6:**
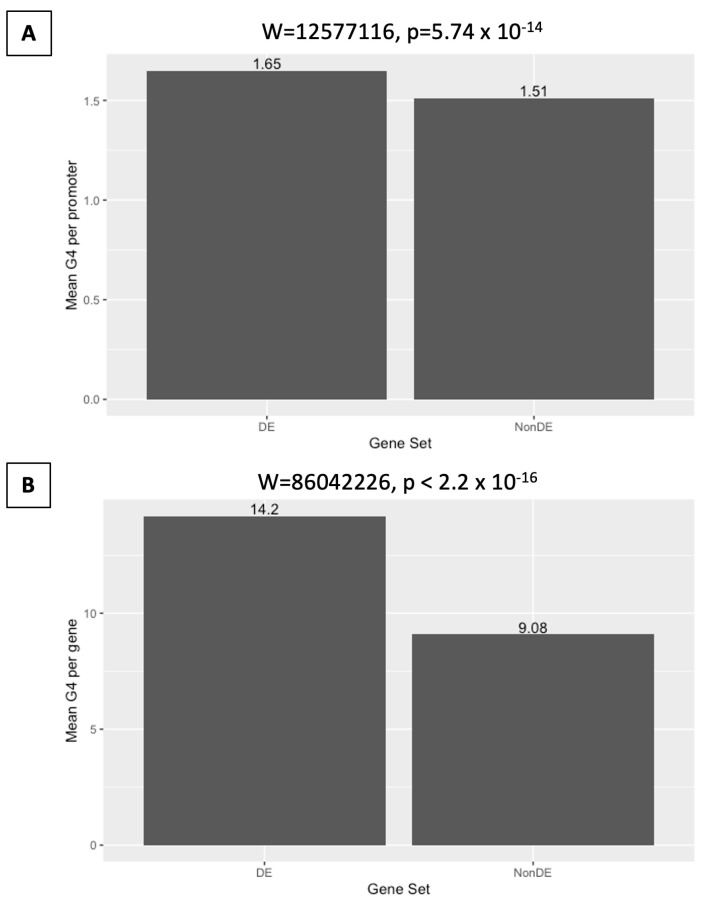
Differentially expressed promoters and genes following *DHX36* KO have significantly more OQs per region than non-differentially expressed genes. (**A**) Mean number of G4s per promoter for differentially and non-differentially expressed genes. (**B**) Mean number of G4s per gene, for differentially and non-differentially expressed genes.

From the 7410 differentially expressed genes, 2682 showed at least one OQ present in the defined promoter region (36.2% of DE gene promoters), with a total of 4420 OQs within the 2682 promoters, for an average of 1.65 OQs per promoter. Promoters of differentially expressed genes were significantly more likely to harbor an OQ (hypergeometric test, *p* = 2.71 × 10^−157^) than the genomic background (Figure 5), and had significantly more OQs per promoter (mean = 1.65) than the 8676 non-differentially expressed genes (Figure 6), with at least one promoter OQ (mean = 1.51) being obtained by Wilcoxon Rank-Sum test (W = 12,577,116, *p* = 5.74 × 10^−14^). As only 1130 differentially expressed genes were found with a fold-change of two or greater (2FC) between treatment and control, we found that 433 promoters (38.3%) harbored at least one OQ, and hence 2FC DE gene promoters were significantly more likely to harbor an OQ (hypergeometric test, *p* = 3.63 × 10^−29^) than the genomic background, and contained a total of 727 OQs for an average of 1.68 OQs per promoter, significantly more (W = 2,585,740, *p* = 9.70 × 10^−5)^ than the 10,925 promoters for genes that were not considered to be differentially expressed (1.54 OQs per promoter on average) under this criterion. These data suggest that *DHX36* knockout preferentially impacts the RNA abundance of genes with G4-rich promoters.

### 2.6. Differential Expression on DHX36 Knockout Is Associated with G-Quadruplex Content in Gene Regions

OQs were found within the gene intervals of 27,091 (56.3%) of 48,149 coding and non-coding genes, for a total of 280,899 gene-located OQs, an average of 10.37 OQs per OQ-containing gene. For DE genes between *DHX36* knockout and control cells, 6779 of 7410 had at least one OQ within the gene (91.5%) for a total of 96,482 OQs and an average of 14.2 OQs per OQ-containing gene. Differentially expressed genes were significantly more likely to contain an OQ (hypergeometric test, *p* = 1.72 × 10^−1131^) than the genomic background, (Figure 5) and had significantly more OQs per gene (mean = 14.2) than the 20,312 non-differentially expressed genes. Figure 6 shows that at least one OQ (mean = 9.08) was obtained via the Wilcoxon Rank-Sum test (W = 86,042,226, *p* < 2.2 × 10^−16^), although DE genes with OQs (mean length = 81,209 bp) were also significantly longer than non-DE genes (mean length = 70,181) with OQs (W = 249,715,340, *p* < 2.2 × 10^−16^). These data suggest that *DHX36* knockout preferentially impacts RNA abundance for genes with G4-rich gene regions.

## 3. Discussion

The association of G4s with gene regulatory regions, particularly promoters, indicates a role for G4s in transcriptional control [16,17,18,19]. The stabilization of G4s has been shown, in some cases, to have negative effects on the level of transcription [26,27], suggesting that their unwinding helicases play a role in gene expression, as the formation and processing of G4 DNA structures may serve as a topological gene-regulatory mechanism. Consistent with this, our results show that the loss of a G4-helicase, DHX36, in Jurkat cells has widespread effects on gene expression, as also seen in other studies [36], but with thousands of genes showing increased or decreased expression in KO cells. Our results indicated that the effect sizes were generally small, as the majority of genes showed a fold-change of less than two, and few genes had fold-changes that exceeded two. Given the upregulation of 11 other G4 helicases (Supplementary Table S1: in bold) known from the human genome [38], it is possible that compensatory effects are acting to attenuate large changes in the expression of G4-regulated genes under *DHX36* knockout.

While the effects are generally subtle, *DHX36* knockout may impact gene expression through multiple mechanisms. Previous studies showed that the presence of functionally relevant G4s [27], or an enrichment of G4-forming motifs or the observed G4s in promoter regions, may be associated with impacts on transcription levels. However, based on OQ locations discerned from G4-Seq data, the promoters in our DE gene set were enriched for the presence of at least one OQ and had a few but significantly more OQs in their promoters than genes that did not change their expression in KO cells relative to the wild-type. However, most of the dysregulated genes lacked G4s in their promoter regions. Although gene-level expression analysis, as performed here, is more robust and less likely to lead to false positives [40], genes may contain alternative transcripts with distinct promoters, which are not considered in this analysis. Enhanced replication could make transcript-level DE analysis feasible and reveal additional promoter OQs linked to changes in gene expression. It is also possible that some of these genes were indirectly affected by being downstream of a transcription factor whose dosage was affected by *DHX36* knockout, as hundreds of transcription factors are among the differentially expressed genes, potentially leading to a cascade of effects on gene expression. 

Alternatively, co-transcriptional or post-transcriptional mechanisms might be involved in the regulation of gene expression modulated by G4s and the DHX36 helicase. Previous studies found the highest number of observed quadruplexes in splice sites and 5′ UTRs, where they might have a post-transcriptional regulatory influence [39]. In one study, *DHX36* knockout enhanced mRNA abundance via post-transcriptional mechanisms only, and by enhancing mRNA stability via G4s in UTRs and in the coding sequences in HEK293 cells [36]. Another study in HeLa cells found DHX36 to be associated with polysomes (i.e., actively translated mRNAs) and that DHX36 depletion increased the translation of G4-containing repressive upstream ORFs, thereby reducing coding region translation [37]. Differences in these studies are likely reflective of the different adherent cell lines utilized (HEK293 vs. Hela). Indeed, we did find a marked excess of differentially expressed genes with OQs within the gene region itself, and DE genes also had significantly more OQs than non-differentially expressed genes under helicase knockout in a non-adherent Jurkat cell line. Both factors may be influenced by the fact that genes that were differentially expressed due to loss of DHX36 activity were significantly longer than those that did not change expression.

While we did not collect data on RNA G4 prevalence in KO cells, Guo and Bartel [41] found no increase in RNA G4 formation after *DHX36* deletion in mouse embryonic fibroblasts. There are many alternative G4 helicases in the human genome, including eight other DEAH box helicases [11,38], and eleven G4 helicases were upregulated in *DHX36* KO cells, so it is not entirely clear whether DHX36 gene expression influences are modulated by the enhanced stability of RNA G4 structures. However, the association of RNA G4s with changes in gene expression following the knockout of an important helicase in human cells suggests a change in G4 state [36,37].

From a functional perspective, genes downregulated by *DHX36* knockout seem to lack the functional coherence of upregulated genes, as few GO, DO, or KEGG terms were significantly enriched in the downregulated set. In contrast, the upregulated gene set was significantly enriched for many GO, KEGG, and DO terms, including several cancers. These cancer associations were for genes that were upregulated in *DHX36* KO cells, which included many oncogenes in which G4 stability may be enhanced, indicating that DHX36 activity could typically act to suppress their expression under normal conditions. This contrasts with some single oncogene studies [26,27] in which the stabilization of G4 by other means led to oncogene downregulation. Therefore, the gene regulatory mechanisms of *DHX36* knockout are areas for future elucidation, as is determining whether other aspects of cellular function are also affected.

## 4. Materials and Methods

The creation of *DHX36* knockout Jurkat cell lines was achieved with CRISPR/Cas9-mediated gene editing and validated with PCR, qPCR, and Western blot, as previously described [42]. Cells were cultured in 10 cm cell plates in DMEM supplemented with 10% (*v/v*) bovine growth serum and 1% (*v/v*) antibacterial–antimycotic. For each replicate, RNA was extracted from a separate plate of cells. The extraction of RNA was performed, and libraries for Illumina sequencing were produced with the NEB Next Ultra RNA Library Prep Kit (New England Biolabs, Ipswich, MA, USA) according to the manufacturer’s instructions, for three replicates from knockout and control cell lines. Paired-end reads of 150 bp length were generated from each library by Novogene Corporation Inc. (Sacramento, CA, USA). Raw reads were filtered to remove adapter sequence, reads with more than 10% ambiguous base calls, or reads in which the quality score was less than 5 for more than 50% of the bases in the read. 

STAR 2.5 [43] was used to align cleaned reads to the human GRCh37 reference genome sequence, and HT-Seq 0.6.1 [44] was used to estimate read counts for annotated genes. A differential expression analysis of read counts was performed with DESeq2 [45] with the significance criterion represented by an adjusted *p*-value of <0.05. Transcription factors among differentially expressed genes were identified by comparison with all annotated entries in the TFCat database of human and mouse transcription factors [46], while oncogenes were identified using the COSMIC database [47]. Enrichment for gene and disease ontology terms, and for the Kyoto Encyclopedia of Genes and Genomes (KEGG) categories corresponding to pathways, processes, or phenotypes, were performed using ClusterProfiler with a p-value adjusted by Benjamini–Hochberg correction for multiple tests of <0.05 [48]. 

We defined promoters as regions 1000 bp directly upstream of the transcription start site (one per gene) of protein-coding (21,514) and non-coding (25,117) genes on autosomal (1–22) or sex chromosomes (X, Y) in the GRCh37 reference genome annotation. G4 locations were obtained as the set common to G4-Seq experiments, which stabilized and identified observed G4s (OQs) across the genome with both K^+^ and pyridostatin [39], and OQs localized to promoters or genes were identified by comparing OQ coordinates to promoter or gene coordinates. Only OQs whose coordinates were entirely within the defined promoter or gene region were considered.

The enrichment of promoter OQs in differentially expressed genes relative to the genomic background (all coding and non-coding genes in the Ensembl annotation) was tested using a hypergeometric test. The average number of OQs per promoter for differentially expressed, and non-differentially expressed genes were compared by Wilcoxon Rank-Sum test given the skewed distribution of the number of OQs per promoter.

## Figures and Tables

**Table 1:** For each replicate of control and treatment cell lines, the number of paired-end reads sequenced, the number passing quality control filters, the percent of bases with phred scores of at least 20, the percentage of reads that map to unique genomic locations, and the percentage of reads that map to multiple genomic locations are shown.

Sample Name	Treatment	Raw Reads	Filtered Reads	% Q20	% Uniquely Mapped	% Multi-Mapping
A11_1	*DHX36* KO	25,210,874	24,430,301	98.11	93.58	2.24
A11_2	*DHX36* KO	28,718,452	27,622,299	98.05	93.26	2.26
A11_3	*DHX36* KO	28,926,801	28,216,080	98.16	93.87	2.14
A28_1	Wild-type	27,734,054	27,130,315	98.12	93.59	2.14
A28_2	Wild-type	26,521,784	25,939,496	98.13	93.87	2.14
A28_3	Wild-type	28,081,209	27,206,143	98.23	93.56	2.23

**Table 2:** Significantly enriched disease ontology (DO) terms for genes upregulated in *DHX36* knockout versus control cell lines. Terms with an adjusted *p*-value less than 0.05 after Benjamini-Hochberg correction for multiple tests were considered significantly enriched.

ID	Description	Gene Ratio	Background Ratio	Raw *p*-Value	Adjusted *p*-Value
DOID:4645	retinal cancer	54/1667	113/7405	2.29 × 10^−9^	6.79 × 10^−7^
DOID:5683	hereditary breast ovarian cancer	86/1667	214/7405	3.04 × 10^−9^	6.79 × 10^−7^
DOID:768	retinoblastoma	53/1667	111/7405	3.34 × 10^−9^	6.79 × 10^−7^
DOID:771	retinal cell cancer	53/1667	111/7405	3.34 × 10^−9^	6.79 × 10^−7^
DOID:0060116	sensory system cancer	57/1667	134/7405	1.49 × 10^−7^	2.01 × 10^−5^
DOID:2174	ocular cancer	57/1667	134/7405	1.49 × 10^−7^	2.01 × 10^−5^
DOID:0050736	autosomal dominant disease	123/1667	394/7405	2.56 × 10^−5^	0.00297116
DOID:12704	ataxia telangiectasia	19/1667	37/7405	0.0001142	0.01159172
DOID:1036	ataxia telangiectasia	69/1667	209/7405	0.00026725	0.02411196

## Data Availability

Raw sequencing reads are available at NCBI’s Short Read Archive under accession number SRRXXXXXX.

## Supplementary Materials

The supporting information can be downloaded at: https://www.mdpi.com/article/10.3390/ijms25031753/s1.
